# Identification and targeting of treatment resistant progenitor populations in T-cell Acute Lymphoblastic Leukemia

**DOI:** 10.21203/rs.3.rs-3487715/v1

**Published:** 2023-10-30

**Authors:** Kai Tan, Jason Xu, Changya Chen, Tiffaney Vincent, Petri Pölönen, Jianzhong Hu, Satoshi Yoshimura, Wenbao Yu, Jonathan Sussman, Chia-hui Chen, Elizabeth Li, Caroline Diorio, Rawan Shraim, Haley Newman, Lahari Uppuluri, Alexander Li, Gregory Chen, Shovik Bandyopadhyay, David Wu, Yang-yang Ding, Jessica Xu, Tristan Lim, Miles Hsu, Anusha Thadi, Kyung Jin Ahn, Chi-Yun Wu, Jacqueline Peng, Yusha Sun, Alice Wang, Rushabh Mehta, David Frank, Lauren Meyer, Mignon Loh, Elizabeth Raetz, Zhiguo Chen, Brent Wood, Meenakshi Devidas, Kimberly Dunsmore, Stuart Winter, Ti-Cheng Chang, Gang Wu, Stanley Pounds, Nancy Zhang, William Carroll, Stephen Hunger, Kathrin Bernt, Jun Yang, Charles Mullighan, David Teachey

**Affiliations:** Children’s Hospital of Philadelphia; University of Pennsylvania; The Children’s Hospital of Philadelphia; Children’s Hospital of Philadelphia; St. Jude Children’s Research Hospital; St. Jude Children’s Research Hospital; St. Jude Children’s Research Hospital; Division of Oncology and Center for Childhood Cancer Research, Children’s Hospital of Philadelphia; University of Pennsylvania; Division of Oncology and Center for Childhood Cancer Research, Children’s Hospital of Philadelphia; Divsion of Oncology and Center for Childhood Cancer Research, Children’s Hospital of Philadelphia; Children’s Hospital of Philadelphia; Children’s Hospital of Philadelphia; Children’s Hospital of Philadelphia; Children’s Hospital of Philadelphia; Division of Oncology and Center for Childhood Cancer Research, Children’s Hospital of Philadelphia; University of Pennsylvania; The Children’s Hospital of Philadelphia; Graduate Group in Genomics and Computational Biology, Perelman School of Medicine; Johns Hopkins University School of Medicine; Division of Oncology and Center for Childhood Cancer Research, Children’s Hospital of Philadelphia; Perelman School of Medicine, University of Pennsylvania; Perelman School of Medicine, University of Pennsylvania; Division of Oncology and Center for Childhood Cancer Research, Children’s Hospital of Philadelphia; Division of Oncology and Center for Childhood Cancer Research, Children’s Hospital of Philadelphia; Graduate Group in Genomics and Computational Biology, Perelman School of Medicine; University of Pennsylvania; University of Pennsylvania; Graduate Group in Genomics and Computational Biology, Perelman School of Medicine, University of Pennsylvania; Graduate Group in Cell & Molecular Biolgy, Perelman School of Medicine, University of Pennsylvania; University of Pennsylvania; The Ben Town Center for Childhood Cancer Research, Seattle Children’s Hospital; Seattle Children’s Hospital; NYU Langone Health; University of Florida; Children’s Hospital Los Angeles; St. Jude Children’s Research Hospital; Division of Oncology, University of Virginia Children’s Hospital, Charlottesville; Children’s Minnesota Research Institute; St. Jude Children’s Research Hospital; St Jude Children’s Research Hospital; St. Jude Children’s Research Hospital; University of Pennsylvania; NYU Langone Health; Children’s Hospital of Philadelphia; Children’s Hospital of Philadelphia; St. Jude Children’s Research Hospital; St. Jude Children’s Research Hospital; University of Pennsylvania, Children’s Hospital of Philadelphia

## Abstract

Refractoriness to initial chemotherapy and relapse after remission are the main obstacles to cure in T-cell Acute Lymphoblastic Leukemia (T-ALL). Biomarker guided risk stratification and targeted therapy have the potential to improve outcomes in high-risk T-ALL; however, cellular and genetic factors contributing to treatment resistance remain unknown. Previous bulk genomic studies in T-ALL have implicated tumor heterogeneity as an unexplored mechanism for treatment failure. To link tumor subpopulations with clinical outcome, we created an atlas of healthy pediatric hematopoiesis and applied single-cell multiomic (CITE-seq/snATAC-seq) analysis to a cohort of 40 cases of T-ALL treated on the Children’s Oncology Group AALL0434 clinical trial. The cohort was carefully selected to capture the immunophenotypic diversity of T-ALL, with early T-cell precursor (ETP) and Near/Non-ETP subtypes represented, as well as enriched with both relapsed and treatment refractory cases. Integrated analyses of T-ALL blasts and normal T-cell precursors identified a bone-marrow progenitor-like (BMP-like) leukemia sub-population associated with treatment failure and poor overall survival. The single-cell-derived molecular signature of BMP-like blasts predicted poor outcome across multiple subtypes of T-ALL within two independent patient cohorts using bulk RNA-sequencing data from over 1300 patients. We defined the mutational landscape of BMP-like T-ALL, finding that *NOTCH1* mutations additively drive T-ALL blasts away from the BMP-like state. We transcriptionally matched BMP-like blasts to early thymic seeding progenitors that have low *NR3C1* expression and high stem cell gene expression, corresponding to a corticosteroid and conventional cytotoxic resistant phenotype we observed in *ex vivo* drug screening. To identify novel targets for BMP-like blasts, we performed *in silico* and *in vitro* drug screening against the BMP-like signature and prioritized BMP-like overexpressed cell-surface (CD44, ITGA4, LGALS1) and intracellular proteins (BCL-2, MCL-1, BTK, NF-κB) as candidates for precision targeted therapy. We established patient derived xenograft models of BMP-high and BMP-low leukemias, which revealed vulnerability of BMP-like blasts to apoptosis-inducing agents, TEC-kinase inhibitors, and proteasome inhibitors. Our study establishes the first multi-omic signatures for rapid risk-stratification and targeted treatment of high-risk T-ALL.

Acute lymphoblastic leukemia (ALL) is the most common pediatric cancer and leading cause of pediatric cancer mortality^1^. Outcomes in B-ALL have improved drastically due to optimization of chemotherapy, development of targeted therapies, and genetically-guided risk stratification. In contrast, while outcomes have improved in T-ALL, most patients who relapse are considered low/favorable risk at diagnosis and few targeted therapies have successfully translated into the clinic. Outcomes for patients with relapsed T-ALL are dismal as there are no effective salvage options. Accordingly, T-ALL is a disease where the goal is to use the most effective therapy at initial diagnosis. There is an urgent need to identify biologic risk factors in T-ALL to inform development of targeted therapeutics and enable early identification of high-risk patients who need alternative strategies for cure^2^.

Bulk genomic study of T-ALL has implicated tumor heterogeneity as a possible mechanism of treatment failure; however, the phenotype of treatment-resistant tumor subpopulations remains undefined. Here, we used single-cell multi-omics to map the tumor landscape of >600,000 T-ALL blasts to the full hierarchy of pediatric hematopoiesis. We identify and characterize a chemo/steroid-resistant bone marrow progenitor-like (BMP-like) tumor population shared between high risk patients across the immunophenotypic spectrum of T-ALL. We used single-cell multiomics, large-cohort bulk genomics, and primary patient xenograft models to establish multi-omic signatures for rapid risk assessment and test novel sensitivities of BMP-like blasts which can be leveraged by currently available targeted therapeutics.

## Results

### Subpopulation level overlap of developmental arrest spectra in immunophenotypically distinct T-ALLs

**Figure 1:**
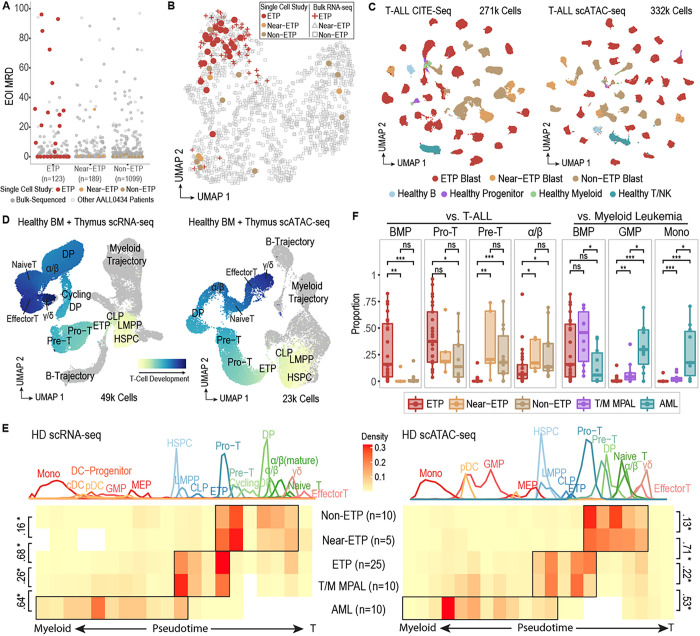
Arrest states of T-ALL subtypes and other acute leukemias in reference to human hematopoiesis. **(a)** Selection of 25 ETP-ALL, 5 Near-ETP ALL, and 10 Non-ETP T-ALL patients from the Children’s Oncology Group AALL0434 cohort (n=1411) based on response to induction therapy (Day 29 MRD). **(b)**UMAP representation of bulk RNA-Seq data from 1335 diagnostic T-ALL samples from COG AALL0434. Each point represents the bulk-RNA-seq transcriptome for one patient. Patients selected for single cell study are indicated by circular points. All ETP patients are colored red. **(c)** UMAP representation of CITE-seq (n=271,603 cells, 271,603 plotted) and scATAC datasets n=332,663 cells; due to size of peak x cell matrix, 60,000 randomly downsampled cells are plotted), colored by level 1 annotations (detailed in Fig. S1). **(d)** UMAP representation of healthy human hematopoiesis development reference trajectories, based on scRNA (left) and scATAC (right) data. Key stages of T-cell development implicated are labeled. **(e)** Arrest state of leukemic cells from 40 T-ALL patients based on projection to healthy scRNA (left) and scATAC (right) reference. D value from two-sample Kolmogorov-Smirnov test is indicated to the side of brackets (*, p<2.2e-16). 10 T/M MPAL and 10 AML patients sequenced using identical assays (Chen et al., in preparation) are included as comparator groups. **(f)** Proportion of ETP-blasts in four key T-cell developmental stages, as compared to other T-ALL patients (left). Proportion of ETP-blasts in three key myeloid developmental stages, as compared to T/M MPAL and AML patients (right). P-values are based on two-sided Mann-Whitney test (*** < 0.001; ** < 0.01, * < 0.05). Results based on scRNA projection are shown. BMP stage encapsulates multipotent progenitors: HSPC/LMPP/CLP/ETP. α/β stage encapsulates all cells that have moved past T-commitment: DP/ α/β / α/β (mature) / Naïve T.

We studied 25 ETP, 5 Near-ETP, and 10 Non-ETP T-ALL patients with varied clinical response from treatment on AALL0434, an international phase 3 randomized Children’s Oncology Group (COG) clinical trial that reported the best published outcomes for children and young adults with T-ALL (Fig. 1a). We selected patients who quickly went into minimal residual disease (MRD) negative remission and were cured (n=16), patients who had intrinsic chemotherapy resistance (refractory, enriched for cases with induction failure; n=10), and patients who relapsed (n=14) (**Supplementary Table 1**). Our samples were broadly representative of AALL0434 cases at the clinical co-variate (**Supplementary Table 2**) and bulk-transcriptomic levels (Fig. 1b), suggesting that our single cell study is well poised to add depth to whole-cohort bulk genomic sequencing across all three immunophenotypic subtypes. We performed Cellular Indexing of Transcriptomes and Epitopes by sequencing (CITE-seq) and single cell Assay for Transposase-Accessible Chromatin (ATAC) sequencing (scATAC-seq) on live cells sorted from cryopreserved diagnostic BM aspirate (n=32) or peripheral blood mononuclear cells (PBMCs, n=8), capturing over 600,000 high-quality cells (**Supplementary Data Fig 1b**-**j**, **Supplementary Table 3**-**4**) across both modalities (Fig. 1c, **Supplementary Data Fig 2a**-**i**). To robustly phenotype our single cell dataset within the context of normal hematopoietic development, we assembled a multiomic reference map of healthy pediatric hematopoiesis, using normal thymus (**Supplementary Table 5**) and bone marrow (**Supplementary Table 6**) tissues collected from children (Fig 1d, **Extended Data Fig 1a**-**h**).

We directly mapped T-ALL blasts to the full hierarchy of human hematopoietic development, overcoming the limitations of using bone marrow restricted references^3^ or mouse derived thymocyte signatures^4^ (Fig. 1e). To assess the integrity of our reference mapping method, we additionally projected scRNA-seq and scATAC-seq data from 10 AML and 10 T-Myeloid Mixed Phenotype Acute Leukemia (T/M MPAL) samples onto our reference, finding that AML blasts projected to the monocytic lineage and T/M MPAL blasts projected to both monocytic and T-cell lineages, as expected. Notably, all five subtypes of leukemia showed a spectrum of developmental arrest states with significant overlap at the subpopulation level. Arrest in a multipotent progenitor-like (HSPC/LMPP) state represented a shared cell state in ETP-ALL, T/M MPAL, and AML; Pro-T-like arrested blasts were shared between T/M MPAL and all three subtypes of T-ALL, and Pre-T-like arrested blasts were shared between Near-ETP and Non-ETP T-ALL (Fig. 1f).

A primary hypothesis for treatment resistance in ETP-ALL has been that ETP-ALL retains myeloid populations that confer resistance to ALL-directed therapy. We found the median frequency of myeloid-projecting blasts (GMP, DC-Progenitor, pDC, cDC, or Mono) to be 0.167% in ETP-ALL patients, in contrast to 16% and 82.5% in T/M MPAL and AML patients, respectively (Fig. 1f). GMP-like and monocytic-like populations comprised <1% of blasts in 18/25 of ETP-ALL patients, and were not detected in 5/25 patients: strongly supporting a lymphoid-progenitor origin of ETP-ALL blasts and use of ALL-directed therapy in ETP-ALL.

Surprisingly, the developmental arrest spectrum of Near-ETP T-ALL was much closer to Non-ETP T-ALL, an unexpected finding given that Near-ETP T-ALL is defined by a stem/myeloid ETP immunophenotype with the exception of high (>75%) CD5 expression. To further assess whether the divergence of ETP and Near-ETP developmental arrest spectra could explain diverging clinical responses of Near-ETP and ETP cases observed within AALL0434 (**Extended Data Fig. 2a**), we performed differentiation expression (DE) and differential chromatin accessibility (DA) analyses (**Supplementary Figure 3a**-**d**).

We observed Near-ETP blasts to have downregulation of stem/myeloid markers (*SPINK2, C1QTNF4, HLA-DRA*) and upregulation of T-cell receptor (TCR) related machinery (*LAT, CD3E, CD28, LCK, PTCRA*) as compared to ETP-ALL blasts, in line with commitment to the T-lineage (**Supplementary Data Fig 3b**). In addition, Near-ETP blasts had increased expression and motif accessibility of TCF7 and LEF1 (**Extended Data Fig. 2b**), two TFs central to T-lineage commitment in healthy thymocytes. Within healthy donor scRNA and scATAC-seq, we observed expression and accessibility of TCF7/LEF1 to rise at the Pro-T stage and peak at the Pre-T stage (**Extended Data Fig. 2c**, **Supplemental Data Fig 4b**), consistent with their known essentiality in T-cell maturation. To ask whether TCF7 and LEF1 have an analogous, T-lineage specific function in T-ALL blasts, we computationally inferred the regulators and targets of TCF7 and LEF1 in ETP, Near-ETP and Non-ETP T-ALL by constructing subtype-specific transcriptional regulatory networks. Our analysis predicts the repression of *TCF7/LEF1* expression by stem-related TFs (ie, MEF2C, IRF1, and LYL1) in ETP-ALL, and activation of TCF7/LEF1 by core TFs of T-cell commitment (ie, BCL11B, SIX6, and TCF7L2) in Near-ETP T-ALL (**Extended Data Fig. 2d**-**e**) and Non-ETP T-ALL (**Supplementary Data Fig 4c**). Our analysis also predicted direct regulation of CD5 expression and TCR-related genes (*LAT, DNTT, MAL, CD3E*) by TCF7/LEF1 in Near-ETP blasts, connecting TCF7/LEF1 regulation to the CD5-bright phenotype observed within diagnostic flow cytometry (**Extended Data Fig. 2e**). Elevated expression of our predicted TCF7/LEF1 regulon was consistently observed in bulk-RNA-seq data of n=110 and n=168 ETP and Near-ETP patients, respectively (**Extended Data Fig. 2f**, **Supplementary Data Fig 4d**). Notably, ETP and Near-ETP patients with higher expression of the TCF7/LEF1 signature had more favorable outcomes within AALL0434 (**Extended Data Fig 2g**-**i**), with TCF7/LEF1 signature having prognostic significance independent of MRD and CNS status in ETP-ALL patients (92.7% vs 79.3% 5-year OS, p = 0.024, **Extended Data Fig. 2h**). Taken together, our results suggest that TCF7/LEF1 activation underlies improved clinical performance within immature (ETP/Near-ETP) T-ALL, highlighting complex and functionally significant transcriptional regulatory circuits that underlie minute, single-marker immunophenotypical differences.

### Treatment resistance in ETP-ALL is associated with a progenitor-like population

High degrees of initial treatment resistance, rather than eventual relapse, contribute to poor outcome in ETP-ALL^5^. Within AALL0434, ETP-ALL patients were 7.1-fold less likely to achieve remission (<5% blasts by morphology on post-induction bone marrow aspirate) after the first month of chemotherapy and more than twice as likely to have MRD detectable by flow cytometry at end of induction (Day 29, EOI) compared to Non-ETP patients. Intrinsic steroid resistance has been suggested to play a central role in treatment resistant ETP-ALL, but the phenotype and mechanism of resistant tumor populations remain unclear.

**Figure 2:**
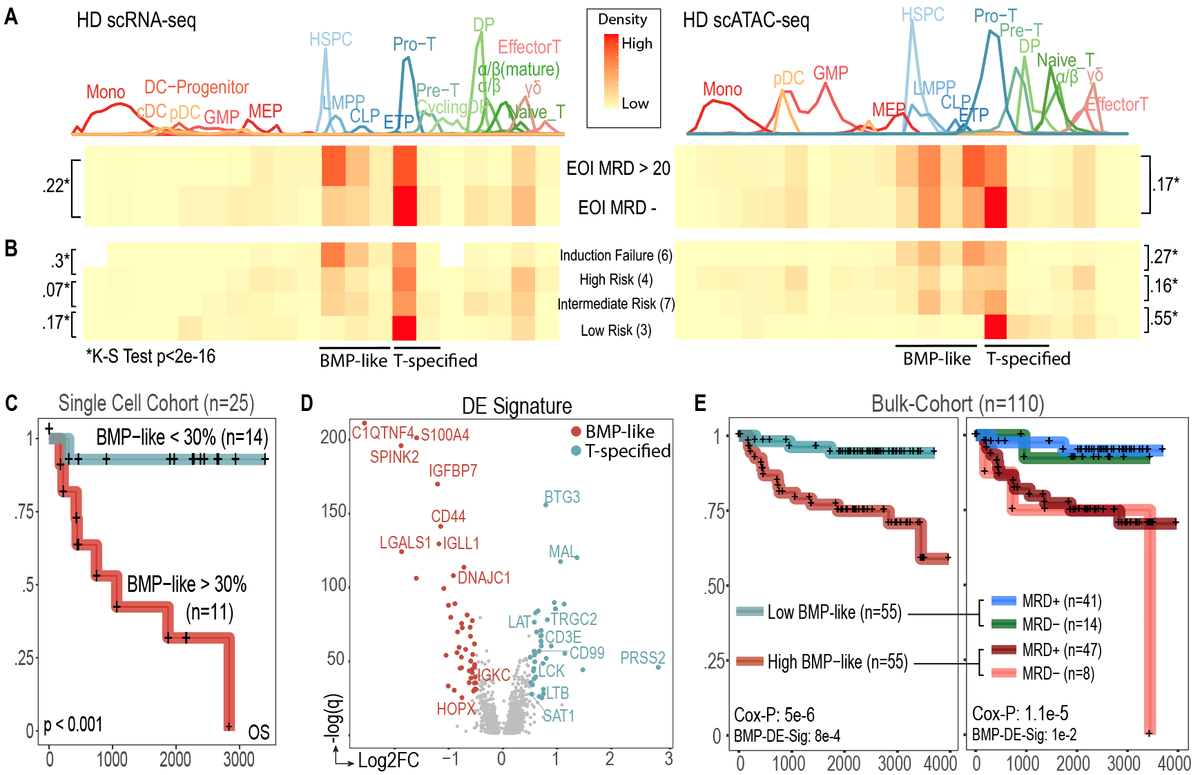
Treatment Resistance in ETP-ALL is associated with a progenitor-like population. **(a-b)** Arrest state of leukemic cells from High MRD and MRD-Negative ETP-ALL patients based on projection to healthy scRNA (left) and scATAC (right) reference trajectory. A, Proportion ranges from 0–0.3. b, Proportion ranges from 0–0.5. D values from two-sample Kolmogorov–Smirnov test are indicated by the brackets (*, p< 2.2e-16). © Stratification of n=25 single-cell-sequenced ETP-ALL patients by BMP-like proportion (High: >30%; Low < 30%). **(d)** Differentially expressed genes between BMP-like blasts from non-responding patients and T-specified blasts from responding patients. © Stratification of n=113 ETP patients from AALL0434 using 119-gene DEGs between BMP-like and T-specified blasts obtained in panel d. Prognostic value of the BMP-like-DEG signature in multivariate analysis (with Day 29 MRD and CNS status) is shown below the Cox-proportional hazard log-likelihood p-value. Left: stratification with signature alone; Right: stratification with signature & EOI MRD status.

We enriched our single-cell cohort for treatment refractory ETP cases to identify tumor cell states associated with initial treatment resistance. To this end, we first compared the initial developmental arrest state of 10 ETP patients with high end-induction MRD (EOI MRD >20%) to 10 ETP patients that were EOI MRD negative (<0.1%) (**Supplementary Data Fig. 5a**-**e**). We first asked if treatment response to chemotherapy was simply correlated with the fraction of actively cycling tumor cells. Although we observed a small enrichment of cycling cells in MRD negative patients (20% vs 16%, **Supplementary Data Fig 5f**-**g**), we observed all treatment refractory patients to have significant proportions of cycling cells, prompting us to further investigate whether treatment response could be better explained by differences in cell arrest state. Within scRNA and scATAC-seq data, we observed that ETP patients with EOI MRD > 20% had an enrichment of blasts at HSPC/LMPP/CLP/ETP developmental stages (Fig. 2a, **Supplemental Data Fig. 5h**). Multi-lineage potency is retained^6,7^ in these states, thus, we termed this cell state bone-marrow-progenitor-like (“BMP-like”). In contrast, ETP patients that were MRD-negative had an enrichment of blasts in the Pro-T/Pre-T stage. These states represent specification to the T-lineage and we henceforth refer to them as “T-specified.” We observed the proportion of tumor blasts in BMP-like and T-specified developmental stages to associate with Day 29 centrally assessed risk (Fig. 2a), overall survival (OS; Fig. 2c), and event free survival (EFS; **Supplementary Data Fig. 5i**-**j**) in single-cell sequenced ETP-ALL patients (n=25).

To test if the molecular signatures of BMP-like and T-specified blasts could be used to stratify other ETP patients within AALL0434, we next performed differential expression (**Supplementary Table 7**) and differential chromatin accessibility (**Supplementary Table 8**) analyses to generate cell type specific molecular signatures. We found BMP-like blasts from non-responding ETP patients had high surface protein expression of myeloid and stem cell markers, such as CD33, CD123, HLA-DR, and CD34, and low expression of T-lineage surface protein markers, such as CD3, CD4, CD2, and CD10 (**Supplementary Data Fig. 3a**). The top differentially expressed genes for BMP-like blasts included stem cell markers (*C1QTNF4, CD44, LGALS1, HOPX*), myeloid-lineage (*S100A4, SPINK2*), and B-lineage markers (*IGLL1, IGKC, IGHM*) (Fig. 2d). The top differentially expressed transcription factors in BMP-like blasts included TFs associated with self-renewal^8–10^ (*MEF2C, HOXA3/4/5/6/9/10/11, MEIS1, HHEX, SPI1*) and recovery from genotoxic stress^11^ (*BCL11A*, **Supplementary Data Fig. 6b**). Several of the BMP-like genes involved in self renewal (*MEF2C, HOXA9, FLI1*), or T-cell developmental block^12^ (*SPI1*) also had increased motif accessibility (**Supplementary Data Fig. 6c**-**e**, **Supplementary Table 9**). In contrast, the top differentially expressed genes (DEGs) for T-specified blasts included T-cell differentiation proteins (*MAL*), TCR components (*LAT, CD3E, LCK, TRGC2*), and thymic honing molecules (*CD99*). The top differentially expressed TFs in T-specified blasts included NOTCH targets^13^ (*HES4, HES1*) and core T-cell commitment TFs^14,15^ (*BCL11B, TCF7, RUNX1*). Interestingly, the top DEG (by fold change) in the T-specified state was *PRSS2*, a trypsin gene that lies adjacent to T-cell receptor beta locus and becomes highly expressed as the TCR beta locus prepares for rearrangement.

Previous single cell studies have suggested that a portion of thymic seeding progenitors (TSPs) to be more stem-like than others – retaining similar markers of multipotency seen in BMP-like ETP blasts^16^. In our analysis, we transcriptomically matched BMP-like ETP-ALL blasts to the earliest, most stem-like subset of TSPs (**Extended Data Fig. 3a**-**c**), revealing two putative mechanisms of treatment resistance. Firstly, we found corticosteroid receptor (*NR3C1*) expression to be directly correlated with T-cell differentiation state (**Extended Data Fig. 3d**). In line with the major role of corticosteroids in ALL induction therapy, *NR3C1* expression was highly predictive of EOI MRD (**Extended Data Fig. 3e**). BMP-like blasts had significantly lowered expression of *NR3C1* (**Extended Data Fig. 3d**), rendering them highly resistant (>80-fold increase in IC50) to prednisolone within in-vitro dose response experiments (**Extended Data Fig. 3f**). We also predicted that BMP-like blasts would have high self-renewal capacity, much like TSPs. Indeed, we observed upregulation of leukemic stem-cell related transcriptional programs in BMP-like blasts (**Extended Data Fig. 3g**), associating with >100-fold resistance to T-ALL induction agents within *in vitro* testing (**Extended Data Fig. 3h**).

We applied the BMP-like DEG signature to stratify 110 bulk-sequenced AALL0434 ETP-ALL patients. Our BMP-like DEG signature, composed of 106 genes and 13 TFs (**Supplementary Table 10**) was predictive of 5-year OS (Fig. 2e, left, 66.4% vs 94.3%, 5Y-OS p = 5e-6) and EFS (68.2% vs 94.3%, 5Y-EFS p = 2e-9) independent of Day 29 MRD and EOI CNS disease. Our BMP-like DEG signature provided robust stratification when MRD status was considered as a binary variable (Fig. 2e, right): identifying a subset of EOI MRD negative patients with inferior survival (High BMP-like & MRD negative, 75.0% 5-year OS) and a group of EOI MRD positive patients with favorable survival outcomes (Low BMP-like & MRD+, 94.9% 5-year OS).

### BMP-like and T-specified states are associated with distinct driver mutations

**Figure 3:**
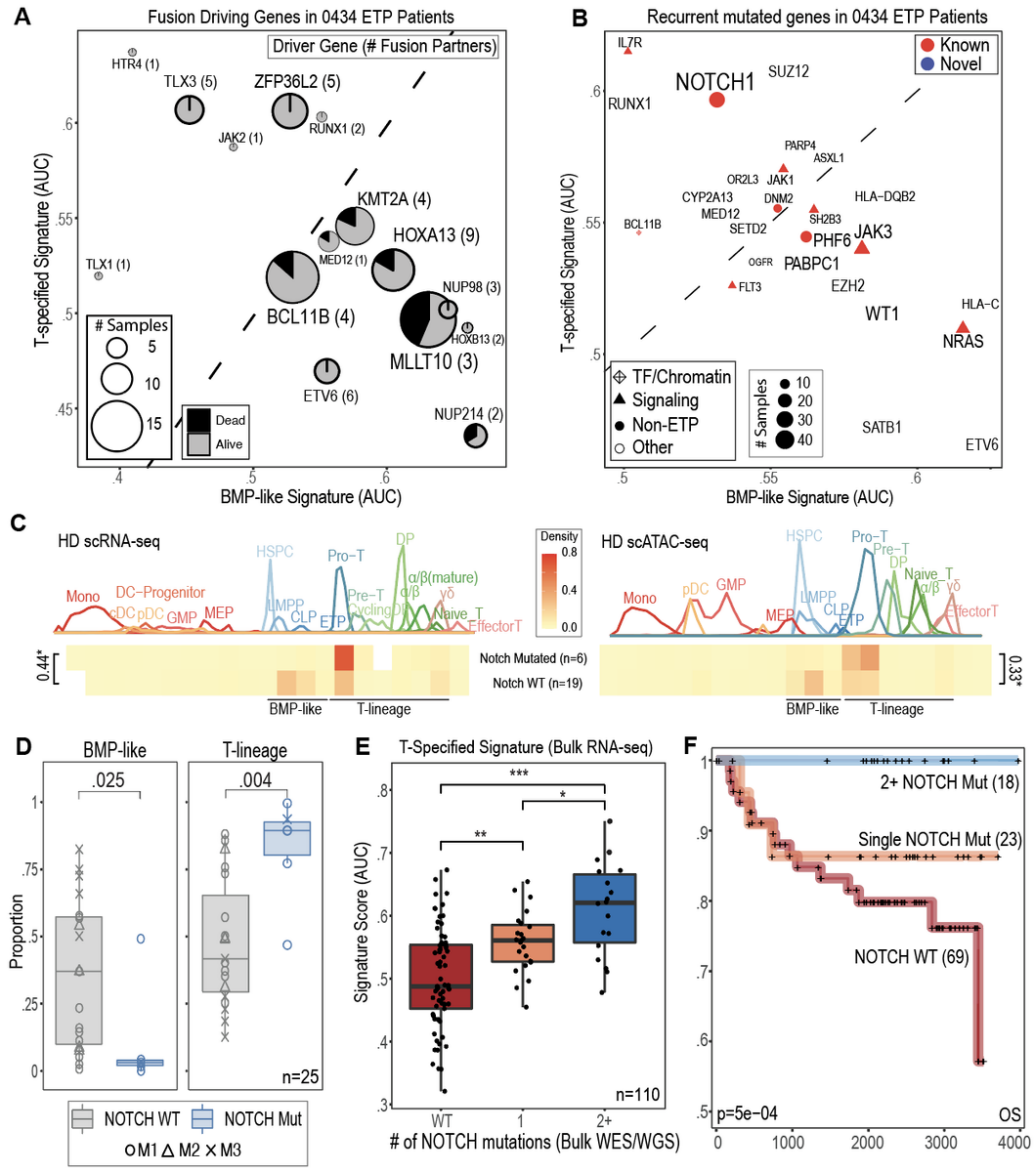
*NOTCH1* mutations are associated with the T-specified rather than BMP-like state. **(a)** recurrent driver fusions (n=16, recurrent in 2+ samples) among 113 bulk-sequenced ETP-ALL patients (Polonen et al) and associated BMP-like and T-specified signature scores. **(b)** Recurrently mutated genes (n=26, recurrent in >5 samples) among 113 bulk-sequenced ETP-ALL patients (Polonen et al) and associated tumor BMP-like and T-specified signature scores. **(c)** Arrest state of leukemic cells from *NOTCH1* WT (n=19) and *NOTCH1*activated (n=6) leukemias based on scRNA and scATAC developmental trajectories. D value from two-sample Kolmogorov-Smirnov test is indicated to the side of brackets (*, p<2.2e-16). **(d)**Proportion of leukemic cells in BMP-like and T-lineage (Pro-T : αβ) cell states in NOTCH1 WT and NOTCH1 mutant in single-cell profiled patients. **(e)** T-specified signature score among 110 bulk-sequenced ETP-ALL patients from AALL0434. Patients are divided into three groups by *NOTCH1* mutation status. P-values from two-sided Mann-Whitney test are shown (* < 0.05; ** < 0.01, ***< 0.001). **(f)** Overall survival of n=113 AALL0434 ETP patients by *NOTCH* mutation status. The p-value for log-likelihood statistic of Cox-proportional hazard test run with Day 29 MRD as a co-variate is shown in the bottom left.

Given the divergent phenotypes observed within BMP-like and T-specified ETP blasts, we next wondered if these two cell states represented acquisition of different driving mutations. To address this question, we leveraged the intersection of single-cell derived phenotypes with structural variant (SV) and single nucleotide variant (SNV) calls (**Supplementary Table 11**) to identify associated drivers of BMP-like and T-specified cell states (Fig. 3a–b, **Supplementary Data Fig. 7a**-**e**). While BMP-like leukemias harbored fusion products known to drive high *HOXA* cluster expression, including *MLLT10, KMT2A, NUP214*, and direct *HOXA::TCR* fusions, T-specified leukemias had fusions to *ZFP36L2* (involved in cell cycle control during T-cell beta selection) and *TLX1/3*. Notably, deaths from disease within the AALL0434 trial (n=110 ETP patients) were all patients with BMP-like associated fusions (Fig. 3a). On the SNV level (Fig. 3b), BMP-like high patients had recurrent mutations in TF and signaling pathways (ie, *JAK3, NRAS, WT1, ETV6* and *SATB1*) while T-specified high patients had mutations in T-lineage regulators (ie, *IL7R, NOTCH1*, and *RUNX1*). Top BMP-like associated SNVs were associated with inferior outcome (**Extended Data Fig. 4a**-**b**, **Supplementary Data Fig. 8a**-**d**), while top T-specified SNVs showed the opposite trend (**Extended Data Fig. 4c**, **Supplementary Data Fig. 8e**-**h**). In line with its essential role in T-lineage commitment, the most recurrently altered gene associated with either cell state was *NOTCH1*. Within our single-cell cohort, we observed that *NOTCH1* mutant patients (n=6) had divergent arrest spectra compared to *NOTCH1* WT (n=19) patients (Fig. 3c), with a notable depletion of BMP-like blasts (Fig. 3d). Interestingly, of patients that were treatment sensitive (EOI MRD <0.1%) with BMP-like associated drivers (*KMT2A* and *MLLT10* fusions), 2 out of 3 harbored tumors with multiple activating *NOTCH1* mutations (**Extended Data Fig. 5a**), suggesting that pan-tumor *NOTCH1* activation can drive differentiation away from the high-risk BMP-like state. This led us to ask whether *NOTCH1* activation represented a universal marker of treatment sensitivity within the larger bulk-sequenced cohort. Of 110 bulk-sequenced ETP-ALL patients, 41 harbored NOTCH1 mutations, with 18 having 2+ mutations (range: 2–5). Patients with 2+ *NOTCH1* mutations had higher T-specified signature scores (Fig. 3e), aligning with an elevated *NOTCH1* variant allele fraction (>50% vs <50%) (**Extended Data Fig. 5b**). Remarkably, all 18 patients with 2+ *NOTCH1* mutations in the bulk-sequenced cohort were alive at last known follow-up, outperforming patients with single *NOTCH1* mutation and *NOTCH1*-wt patients (Fig. 3f).

To study *NOTCH1*-mutant subclones at the single-cell level, we performed genotyping of transcriptomes (GoT) on two patients harboring a total of 7 unique activating *NOTCH1* mutations (**Extended Data Fig. 5a**-**c**, **Supplementary Data Fig. 9a**-**d**). We successfully detected 7/7 *NOTCH1* mutations in scRNA-seq libraries, corroborating bulk-derived variant calls (**Extended Data Fig. 5d**, **Supplementary Data Fig. 9e**-**g**). In strong support of the observations gleaned from bulk data, we found that *NOTCH1*-mutant cells were predominantly in the T-specified, rather than BMP-like cell state (Extended Data **Fig. 5e**). Remarkably, we discovered hundreds of leukemic blasts that carry 2 co-occurring *NOTCH1* mutations, likely resulting from selection for *NOTCH1* mutation in distinct alleles (**Extended Data Fig. 5f**). We found blasts with 2 unique mutations to have the highest expression of T-specified genes and lowest expression of BMP-like genes (**Extended Data Fig. 5g**), revealing a direct connection between *NOTCH1* mutation dosage and the T-specified cell state (**Extended Data Fig. 5h**). Taken together, these data cement *NOTCH1* mutation status as critical biomarker for response to conventional therapy and offer high-resolution insight as to how *NOTCH1* mutations alter T-ALL cell state.

### BMP-like sub-populations predict outcome in immunophenotypically mature T-ALL

Relapsed/refractory T-ALL is nearly universally fatal due to acquired chemo-resistance and lack of targeted therapy options. Given that BMP-like blasts are highly resistant to conventional T-ALL therapy, we wondered if an analogous, less differentiated subpopulation could be responsible for treatment resistance and relapse in Non-ETP T-ALL. Analysis of bulk-RNA sequencing data from the AALL0434 Non-ETP cohort support this hypothesis, with differential expression between 355 MRD+ and 714 MRD-Non-ETP T-ALL cases (**Supplementary Table 12**) revealing gross differences in differentiation state (**Supplementary Data Fig. 10a**). Within this analysis, MRD-negative patients overexpressed markers of the α/β stage (*CD1B, CD1E, MAL, CD8A, PTCRA, RAG1/2*), while MRD+ patients expressed immature forms of the TCR (*TRGC1/2*) and stem-related TFs (*HHEX, LYL1*). We utilized single-cell multiomics data to determine whether these differences were mediated by cell state or cell proportion differences.

**Figure 4:**
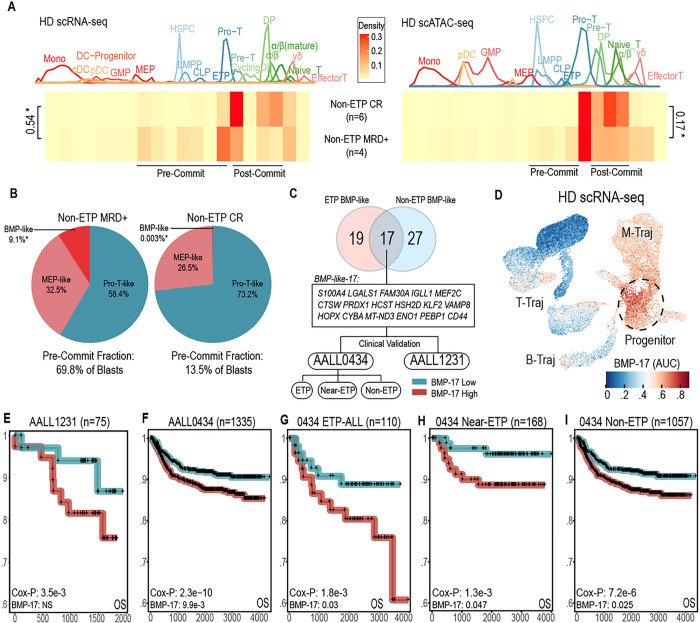
A consensus, 17-gene BMP-like signature predicts overall survival across all subtypes of T-ALL. **(a)** Arrest state of leukemic cells from CR and MRD+ Non-ETP-ALL patients based on projection to healthy scRNA (left) and scATAC (right) reference trajectory. **(b)** Arrest state of pre-committed Non-ETP blasts in CR and MRD+ patients. BMP-like encapsulates all cells that possess multipotent potential: HSPC/LMPP/CLP/ETP. **(c)**Overlap of ETP BMP-like and Non-ETP BMP-like DEGs to create consensus signature for risk stratification in AALL0434 (fully sequenced) and AALL1231 (partially sequenced). BMP-like DEGs were filtered for mean Log2FC > 0.9 between ETP and Non-ETP comparisons. **(d)** BMP-17 signature score within BM/thymus scRNA-seq reference. Multipotent BM progenitor populations with high BMP-17 AUC are circled. (**e-i)**, Kaplan-Meier plot showing overall survival of bulk-RNA-sequenced T-ALL patients in AALL0434 and AALL1231 binarized using the BMP-like-17 signature. Prognostic value of the BMP-like-17 signature in multivariate analysis (with Day 29 MRD and CNS status) is shown below the Cox-proportional hazard log-likelihood p-value.

We stratified our 10 Non-ETP patients into two groups: 6 patients with complete response (CR; EOI MRD-negative), and 4 patients that were EOI MRD+ (EOI MRD > 0.1%). Within both scRNA and scATAC projections to reference, we observed that non-CR patients had an enrichment of cell states prior to T-cell commitment (Pro-T, CLP, LMPP, MEP, HSPC), while CR patients had an enrichment of cells in post-commitment states (DP, α/β) (Fig. 4a, **Supplementary Data Fig. 10b**). Since pre-committed blasts represent a continuum of cell states from BMP-like to Pro-T-like, we compared the distribution of pre-committed blasts in CR patients with non-CR patients. We found that MRD+ patients harbored a strong enrichment of BMP-like blasts, which were near absent in CR patients (Fig. 4b). We next performed differential expression analysis and generated signatures (**Supplementary Table 13**-**14**) for both pre-committed non-ETP blasts and BMP-like non-ETP blasts (**Supplementary Data Fig. 10c**-**d**). We found that these signatures were both able to stratify Non-ETP patients from two independent COG trials (AALL0434, full cohort sequenced; AALL1231, partially sequenced cohort) independent of MRD, with the BMP-like signature having slightly better stratification in both cases (**Supplementary Data Fig. 10e**-**f**).

We next intersected the molecular signature of BMP-like blasts obtained from ETP-ALL and Non-ETP T-ALL patients, revealing a shared BMP-like gene set (“BMP-17”) composed of 17 marker genes (Fig. 4c, **Supplementary Table 15**) typically expressed in stem, myeloid, and B-cell progenitors (Fig. 4d, **Extended Data Fig. 6a**). We applied BMP-17 to five different clinical scenarios for risk stratification of T-ALL patients (Fig 4c), observing robust risk-stratification in all instances (Fig. 4e–i). BMP-17 powered risk stratification within the smaller, partially sequenced AALL1231 cohort (Fig. 4e), and was prognostic independent of EOI MRD and EOI CNS status within the fully sequenced AALL0434 cohort (Fig. 4f), including when patients were stratified by ETP status (Fig. 4g–i). Using single-cell antibody derived tag (ADT) values obtained from CITE-seq, we next determined the surface immunophenotype of BMP-like blasts, revealing a 9-marker phenotype (“BMP-surface-9”, **Extended Data Fig. 7a**, **Supplementary Table 16**) that reflected similar lineage-aberrancy (**Extended Data Fig. 6b**) observed with BMP-17. Because our ADT panel detects of 22 surface epitopes used in the flow cytometric diagnosis of T-ALL, we reasoned that the surface phenotype of BMP-like blasts could be highly clinically actionable. Risk stratification using BMP-surface-9 genes in bulk-RNA-sequenced patients lend strong support to this hypothesis, with RNA expression of BMP-surface-9 genes robustly powering risk stratification within the AALL0434 and AALL1231 T-ALL cohorts (**Extended Data Fig. 7b**-**e**). We additionally validated the BMP-surface-9 in n=99 AALL0434 diagnostic flow cytometry cases (**Extended Data Fig. 7g**-**i**), finding robust correlation of clinically used surface markers with the BMP-17 gene signature. We next turned out attention towards non-pediatric T-ALL, which is enriched for both treatment refractory cases and ETP phenotype. To test whether our BMP-subpopulation signatures could be extended to scenarios of non-pediatric T-ALL, we applied BMP-17 to young-adult (age > 18) cases treated on AALL0434: successfully isolating a subset of high-BMP cases (**Extended Data Fig. 8a**-**c**) with high rates of EOI MRD/induction failure (**Extended Data Fig. 8d**-**e**) and reduced OS and EFS (**Extended Data Fig. 8f**). These results highlight the prognostic utility of immunophenotype-based detection of high-risk BMP-like blasts, revealing a novel, subpopulation-based use case for clinical flow cytometry data. Additionally, our results highlight the potential prognostic utility of BMP-like blasts in both pediatric and non-pediatric settings and underline the utility of single-cell methods to uncover mechanisms of treatment resistance seen exclusively at sub-population level.

### *In silico* and *in vitro* drug screening identify *BCL2* and *BTK* dependency in BMP-like blasts

**Figure 5:**
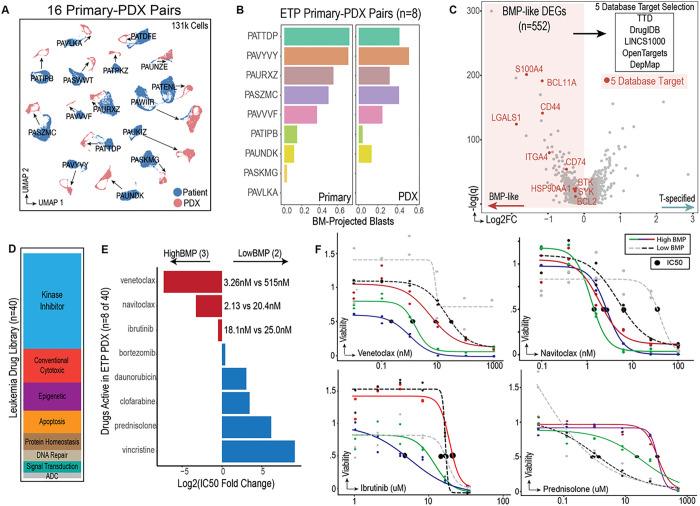
Nomination and pre-clinical validation of targeted therapy against BMP-like blasts. **(a)** UMAP representation of 16 primary patient samples and corresponding PDX models profiled using scRNA-seq. PDX engrafted blasts are connected to their primary sample via arrows. **(b)** proportion of BM projected blasts (HSPC/LMPP/CLP) in 8 patient-PDX pairs. Samples are ordered by proportion of BM-projected blasts in the primary sample (left). Patients PATTDP, PAVYVY, PASZMC, PAVVVF had induction failure with BMP-like blasts > 25%. **(c)** Computational screening approach used to identify targetable genes within BMP-like blasts. 552 BMP-like blast specific DEGs (FDR < 0.05) were overlapped with 3 different drug target databases (TTD, DrugIDB, OpenTargets), LINCS1000 (a transcriptomic-based compound screening database), and DepMap (cancer cell line crispr-dependency database). Each gene was assigned a database score (+1 for each database) and differential expression score (ranging from 0–3). The top 10 targets by aggregate score are highlighted in red. **(d)** A panel of 40 drugs (left) was tested on PDX engrafted blasts from 5 T-ALL patients (2 high BMP-like and high MRD, 1 high BMP-like with relapse, 1 low BMP-like MRD- ETP, 1 low BMP-like MRD- Non-ETP). **(e)** Relative activity of drugs active in High BMP-like vs Low-BMP-like leukemias. **(f)** Dose response curves for nominated therapeutics that showed differential activity in high-BMP-like vs low-BMP-like leukemias. Response to prednisolone is shown as a comparison.

The universal existence of BMP-like populations across treatment refractory T-ALL cases prompted us to develop a pipeline for development of BMP-like directed targeted therapy. To support the modeling of BMP-like therapy response, we first expanded blasts from 22 single-cell sequenced patients in NSG mice (**Extended Data Fig. 9a**-**c**). Single cell RNA-sequencing on engrafted blasts from 16 patients indicated strong retention of patient-specific features, with most PDX-engrafted blasts projecting directly adjacent to their patient source (Fig. 5a). In addition, we found that BMP-high patients maintain BMP-high phenotypes post engraftment (Fig. 5b), aligning with our observation that BMP-high leukemias engrafted at slightly higher efficiency (**Extended Data Fig. 9d**-**e**).

Having identified a putative driver of resistance in these patients, we next considered how to leverage this as a potential therapeutic vulnerability. We thus performed computational screening for targets that would be druggable and specific to BMP-like blasts from treatment resistant patients. We queried 552 BMP-like specific genes against three drug target databases (TTD, DrugIDB, OpenTargets, **Extended Data Fig. 10a**), one transcriptomic-based compound screening database (LINCS1000, **Extended Data Fig. 10b**), and one cancer gene vulnerability database (DepMap, **Extended Data Fig. 10c**). The consensus results from our 5-database approach (**Supplementary Table 17**, **Extended Data Fig. 10d**) nominated four druggable surface proteins (CD44, LGALS1, ITGA4, CD74), three homeostatic enzymes (S100A4, BCL2, HSP90), two signal transduction molecules (SYK, BTK), and one transcription factor (BCL11A) (Fig. 5c).

To test these computational predictions, we performed *in vitro* drug screening using an established panel of 40 leukemia active drugs (Fig. 5d, **Supplementary Table 18**). PDX-expanded blasts from 5 patients were screened using a stromal cell co-culture system and dose response curves were generated for each compound (**Supplementary Table 19**). Out of 40 compounds, 9 compounds were active in all PDX models (**Extended Data Fig. 10e**). These compounds had different activity across BMP-high patients (n=3) and BMP-low patients (n=2). While BMP-high/MRD+ patients had increased sensitivity to venetoclax, navitoclax, and ibrutinib, BMP-low/MRD− patients were more sensitive to conventional cytotoxics (prednisolone, clofarabine, daunorubicin) (Fig. 5e–f). Lowered expression of the steroid receptor (**Supplementary Data Fig. 5d**), and upregulation of leukemic stem-cell related transcriptional programs (**Supplementary Data Fig. 5g**) in BMP-like blasts provide potential explanations for >80-fold and >100-fold resistance to corticosteroids and chemotherapy, respectively.

BCL2 (5 database hit) and BTK (4 database hit) were top predicted BMP-like vulnerabilities based on our 5-database approach (**Extended Data Fig. 10d**). The strong *in vitro* activity of apoptosis-inducing agents against BMP-like blasts prompted us to initiate *in vivo* efficacy studies in BMP-high (PATTDP, n=6) and BMP-low (PAUNDK, n=9) PDX models (**Extended Data Fig 9f**). After 4 weeks of venetoclax treatment, we performed flow cytometry to quantify the residual leukemic burden in both bone marrow and spleen (**Extended Data Fig. 9f**). In the peripheral blood, venetoclax treatment resulted in halting of disease progression in BMP-low models compared to control (**Supplementary Data Fig. 11a**-**c**). However, after the conclusion of the study, BMP-low PDX models still harbored significant residual disease within both the bone marrow (>38% blasts) and spleen (>4% blasts) (**Extended Data Fig. 9g**, **right,** **Supplementary Data Fig 12a**-**b**). In contrast, venetoclax treatment resulted in robust clearance of disease in BMP-high PDX (**Extended Data Fig. 9g**, **left,** **Supplementary Data Fig. 11d**-**f**), with reduction of blasts to beyond our limit of detection (<0.01%) the majority of PDX (**Supplementary Table 20**). We found these robust responses to be present in both the bone marrow and spleen (**Extended Data Fig. 9h**) and easily detected by hCD7+/hCD45+ immunophenotypic profiling (**Supplementary Data Fig. 13a**-**b**). Our results support a new treatment paradigm for T-ALL involving: (1) early genetic screening for the chemotherapy-refractory BMP-like phenotype and (2) clinical testing of BCL2 inhibitors in refractory BMP-like T-ALL.

## Discussion

Our study reports the first comprehensive mapping of T-ALL to healthy human hematopoiesis. We report the surprising discovery that T-cell leukemias differing drastically by bulk-immunophenotype are linked at the subpopulation level. Our integrated analysis identifies a shared BMP-like population tightly associated with treatment failure in ETP, Near-ETP, and Non-ETP T-ALL. This subpopulation can represent <5% of blasts at diagnosis, illustrating the limitations of current bulk-level tumor classification schemes. Interestingly, BMP-like blasts are also found in myeloid and mixed phenotypic leukemias, raising the possibility of one common cell of origin. Evidence supporting this includes an enrichment of TF and signaling gene mutations within BMP-like T-ALL blasts (a mutational spectrum shared with myeloid leukemias), non-T lineage marker expression, and shared drug sensitivity profiles with myeloid leukemia stem cells. The unique opportunity to intersect our single-cell data with large-cohort bulk WES/WGS data allowed us to the associate BMP-like phenotype and genotype at subclonal resolution. Our data implicating the inverse relationship between *NOTCH1* activation and the BMP-like state represents a potential therapeutic strategy in T-ALL and provide rationale for *NOTCH1* activation, rather than inhibition, to treat high-risk T-ALL. Although our study is heavily focused on pediatric T-ALL, our findings are perhaps even more relevant in the adult setting, where the ETP phenotype represents up to 52% of cases and 5-year survival rates are <50%. Detection of high-risk cases within young adults treated on AALL0434 using BMP-like signatures supports the hypothesis that BMP-like blast mediated treatment failure extends beyond pediatric T-ALL cases. Interestingly, within ECOG2993, one of the largest adult T-ALL cohorts genomically studied to date, *NOTCH1*-mutation status conferred a significant survival advantage, further supporting a common mechanism of treatment resistance mediated by *NOTCH1*-wt, BMP-like subpopulations. Further genomic and experimental studies should be rapidly explored to confirm the phenotype and prognostic utility of BMP-like blasts within adult T-ALL cases. Finally, our single-cell multiomic reference maps of human hematopoiesis represents a valuable resource to further delineate the impact of developmental heterogeneity in human leukemia. Utilization of these references maps within five subtypes of acute leukemia underlined a BMP-like arrest state shared between lymphoid, myeloid, and mixed phenotype leukemic disease, highlighting opportunity for further study. For instance, these reference maps and gene signatures could be utilized to study tumor evolution in the context of relapsed tumors and serial samples. Collectively, our study identifies a rare but clinically important BMP-like subpopulation which represents a promising therapeutic target in the continuing effort to rescue relapsed/refractory T-ALL patients from their currently dismal prognosis.Single-cell approaches on a carefully selected cohort were uniquely powered to isolate the BMP-like gene signature for risk stratification and therapeutic targeting: illustrating a prime example where high-resolution single-cell analyses are needed to supplement high-throughput bulk genomic approaches for understanding clinically relevant tumor biology.

## Materials and Methods

### AALL0434 Patient Identification and Clinical Annotation

Children’s Oncology Group (COG) studies AALL0434 (NCT04408005) and AALL1231 (02112916) were approved by the NCI Cancer Evaluation and Therapeutic Program (CTEP), the FDA, the Pediatric Central Institutional Review Board (IRB), and by local IRBs at all participating centers. Written informed consent/assent was obtained from study participants and, when appropriate, their legal guardians, in accordance with the Declaration of Helsinki.

Secondary genomic studies were approved by the Children’s Hospital of Philadelphia (CHOP) IRB. 40 cases from AALL0434 (Supplementary Table 1) and 8 healthy thymus and bone-marrow controls (Supplementary Table 5–6) were selected for single-cell study.

Within AALL0434, ETP status was centrally assessed in diagnostic bone marrow (BM) or peripheral blood (PB) samples by 8–9 color multiparameter flow cytometry.^26^ ETP was defined as having lymphoblasts that were CD8negative and CD1anegative (<5% positive), weakly expressed CD5 (either < 75% positive or median intensity more than 1 log less than mature T cells), and expressed one or more myeloid or stem cell markers (>25% positive) including CD13, CD33, CD34, CD117 or HLA-DR.^13^ Subjects meeting the ETP immunophenotypic criteria, but with stronger expression of CD5 were classified as Near-ETP. Subjects with neither ETP nor Near-ETP were defined as Non-ETP.

MRD was assessed using 8 and 9 color flow cytometry and was performed using established methods at a COG flow cytometry reference laboratory (University of Washington-BLW; or Johns Hopkins University-MJB).

### Processing of T-ALL diagnosis samples for single cell assays and injection to PDX

Peripheral blood (PB) or bone marrow aspirate (BM) samples were thawed at 37°C, treated with 1/10 volume 1mg/mL DNase I (Sigma, Cat #: D4513) for 90 sec at 37°C, resuspended in 10mL IMDM + 2% FBS and centrifuged (1200 rpm 5 min). Samples were retreated with DNase I and resuspended in FACS buffer (Ca^++^ and Mg^++^ free PBS + 1% BSA). Cell number and viability were recorded using Countess II cell counter (Invitrogen). >1 million live cells were aliquoted for tail vein injection into NSG mice, with the remaining stained with DAPI (Invitrogen, Cat #: D1306) and subject to FACS sorting (FACSAria Fusion, BD).

### scRNA/CITE-seq library preparation

FACS sorted DAPI-live cells were centrifuged and resuspended in Cell Staining Buffer (BioLegend, Cat #: 420201) at 45uL/million cells. Cells were blocked with Human TruStain FcX (BioLegend, Cat #: 422301) at 5uL/million cells (4°C, 15 min). After blocking, cells were stained with a TotalSeq-A antibody cocktail (see Supplementary Methods; 30 min, 4°C). Cells were washed 3x using Cell Staining Buffer (BioLegend, Cat #: 420201) and resuspended in PBS+0.04%BSA. Cells were counted using Countess II cell counter. 17,000 cells per sample were then loaded onto 10x Genomics Chromium controller and processed with the Chromium NEXT GEM Single Cell 3’ reagent kits V3.1. GEX libraries were constructed using 10x Genomics library preparation kit followed the instruction. ADT libraries were constructed using KAPA HiFi HotStart ReadyMix kit (Kapa Biosystems, Cat #: KK2601). The following program was used for ADT library PCR: 98°C for 2min, 14–15 cycles of (98°C for 20 sec, 60°C for 30 sec, 72°C for 20 sec), 72°C for 5 min, and hold at 4°C. Library quality was checked using Agilent High Sensitivity DNA kit (Agilent, Cat #: 5067–4626) and Bioanalyzer 2100. Libraries were quantified using dsDNA High-Sensitivity (HS) assay kit (Invitrogen, Cat #: Q33231) on Qubit fluorometer and quantified using the qPCR-based KAPA quantification kit (Kapa Biosystems, Cat #: KK4844). Libraries were sequenced on an Illumina Nova-Seq 6000 with 28:8:0:87 paired-end format.

### scATAC-seq library preparation

DAPI-live cells were centrifuged at 300*g* (5 min at 4°C) then mixed in 45uL lysis buffer and incubated (3 min on ice). Next, 50μL of prechilled wash buffer was added without mixing and centrifuged immediately at 300*g* (5 min at 4°C). 95μL of supernatant was discarded, 45μL diluted nuclei buffer (10x Genomics) was added, and sample was centrifuged (300*g*; 5 min at 4°C). The nuclei pellet was then resuspended in 7μL prechilled diluted nuclei buffer, and nuclei concentration was determined using a Countess II cell counter. 7,000–20,000 nuclei were used for the transposition reaction in bulk, and then loaded onto the 10x Genomics Chromium controller and processed with the Chromium NEXT GEM Single Cell ATAC Reagent Kit V1.1. Library quality was checked using Agilent High Sensitivity DNA Kit and Bioanalyzer 2100. Libraries were quantified using dsDNA High-Sensitivity (HS) assay kit on Qubit fluorometer and the qPCR-based KAPA Quantification Kit. Libraries were sequenced on an Illumina Nova-Seq 6000 with 49:8:16:49 paired-end format.

### Expansion of T-ALL blasts in PDX and post-engraftment scRNA/CITE-seq

PDX expanded blasts were harvested from spleen or bone marrow. Frozen samples were thawed (37°C) and resuspended in IMDM + 2% FBS and treated with DNase I twice. Cells were gently washed 2x with RPMI, resuspended in flow buffer, stained with DAPI and anti-human CD45 antibody (BD Pharmingen, Cat #: 555485) and subject to FACS sorting (FACSAria Fusion, BD). DAPI-hCD45+ sorted cells were stained with 10x Genomics 3’ CellPlex multiplexing solution, washed 3x, and immediately processed using the 10x Genomics Chromium controller and the Chromium NEXT GEM Single Cell 3′ reagent kits (V3.1). 3′ GEX libraries were constructed using 10x Genomics library preparation kit. CellPlex libraries were constructed using 10x Genomics 3’ CellPlex kit. Library quality was checked using Agilent High Sensitivity DNA kit and Bioanalyzer 2100. Libraries were quantified using dsDNA High-Sensitivity (HS) assay kit on Qubit fluorometer and the qPCR-based KAPA quantification kit. Libraries were sequenced on an Illumina Nova-Seq 6000 with 28:8:0:87 paired-end format.

### CD34+ progenitor isolation from infant/pediatric thymus

Pediatric thymi were obtained and used according to and with the approval of the CHOP IRB. Thymus tissue was mechanically disrupted and treated with liberase (0.2mg/mL Roche; 30 min at 37°C) with intermittent shaking, as previously described^17^. Thymocytes were resuspended into flow buffer, sorted into DAPI-Lin-CD34+CD1A− fractions and subject to scRNA-seq and scATAC-seq. The antibodies used for cell sorting described above were listed in Supplementary Methods.

### Projection of patient scRNA and scATAC data onto healthy reference trajectory

Patient derived cells were projected onto the healthy reference trajectory using the MapQuery function in Seurat v4.0.5^18^, which assigns (1) predicted reference UMAP coordinates (2) nearest reference cell-type label, and (3) confidence score for nearest reference cell-type labels. In addition to default cell type label transfer, predicted T-cell trajectory pseudotime values and Myeloid-trajectory pseudotime values were transferred. To order myeloid development and T-lineage development onto a single trajectory, pseudotime values underwent the same transformations as described for healthy reference data.

For scRNA-seq data, the UMAP model was constructed with the top 25 principal components and the min.dist parameter of 0.5 using uwot 0.1.10 function. Next, patient and healthy control data were co-embedded into a low-dimensional space using the default anchor-based CCA method in Seurat 4.0.5 (30 dimensions, 2,000 anchor features), and cell type label transfer was performed on a sample-by-sample basis using the TransferData function.

For scATAC-seq data, the UMAP model was constructed using uwot 0.1.10 with dimensions 2 to10 from latent semantic index based reduction (LSI) and the min.dist parameter of 0.5. Prior to anchor-based integration and label transfer, peaks from patient and healthy reference data were merged using the mergePeaks module from scATAC-pro^19^, and peak-by-cell matrices with merged peaks were reconstructed for each patient with the scATAC-pro reConstMtx module. This allowed for patient and healthy control data to be co-embedded into a low-dimensional space using the default anchor-based CCA method in Seurat 4.0.5 (30 dimensions, 2,000 anchor features), and cell type label transfer to be performed on a sample-by-sample basis using the TransferData function.

### Quantification of developmental arrest state

Two independent visualizations – heatmap (plotting the distribution of blasts along pseudotime) and boxplot (plotting the proportion of blasts in any defined cell state) – were used to delineate the arrest profile of T-ALL blasts. T-ALL blasts were subset from non-malignant cells, leaving 250,911 and 261,500 cells for analysis in CITE-seq and scATAC-seq datasets, respectively.

Heatmaps of T-ALL blast arrest were used to visualize the overall arrest state of blasts and were plotted with the following parameters: predicted pseudotime (myeloid from −1 to 0; T from 0 to 1) as the x-axis, comparator variable (ETP status, MRD status, risk group, etc) as the y-axis, proportion as the fill, 20 bins. Above each heatmap, cell types from healthy donor scRNA/scATAC-seq trajectories were plotted with the following parameters: pseudotime (myeloid from −1 to 0; T from 0 to 1) as the x-axis, frequency as the y axis. Statistical comparisons in overall arrest state were made using two-sample Kolmogorov-Smirnov (KS) test, as previously described^20^.

Boxplots were used to inspect patient-level arrest proportions in key cell states learned from heatmap visualizations. Each dot represents one patient and each panel one cell state. Patients were dichotomized by comparator variable (ETP status, MRD status, OS, EFS, etc). Statistical comparisons were made using Wilcoxon rank-sum test, unless otherwise specified in the figure legend.

### AALL0434 ETP-ALL stratification using BMP-119 signature

BMP-like and T-specified DEGs were stringently filtered using FDR < 0.001, average Log2FC > 0.5 cutoffs, leaving 66 BMP-like DEGs and 53 T-specified DEGs. Z-score based signature scoring was performed on 113 bulk-sequenced ETP-ALL patients with BMP-like DEGs as positive features, and T-specified DEGs as negative features. For each patient, the mean T-specified-feature Z-score was subtracted from the mean BMP-like-feature Z-score, with a score of >0 being interpreted as BMP-like > T-specified. Association of EFS and OS with BMP-like signature was assessed by two methods. First, patients were binarized by EFS/OS status (event vs no event; dead vs alive) and signature scores compared using Wilcoxon rank-sum test. Second, patients were binarized by BMP-like signature score (BMP-like > T-specified vs T-specified > BMP-like) and OS and EFS compared using the Cox Proportional Hazard model with Day 29 MRD and CNS status taken as covariates using the survfit function from survival 3.2–13: *survfit(Surv(time.survival, status.survival) ~ high.BMP + D29.MRD + D29.CNS.Status.*

### Integration of single cell signatures with bulk WES/WGS/RNA-seq derived mutation calls

Bulk-RNAseq data for n=110 ETP samples with corresponding WES/WGS mutation calls were scored using 66 BMP-like DEGs and 53 T-specified DEGs using AUCell^21^ v1.12.0. For 1,490 mutant genes in 110 ETP samples, the number of samples carrying mutations was quantified and the mean BMP-like AUC and T-specified AUC were calculated. Mutant genes observed in >= 5 samples and mean VAF > 0.05 were plotted for visualization. Classification of genes were derived from previous bulk genomics study on ETP-ALL^22^.

For fusion drivers (15 fusion drivers out of 49 unique fusions) in 110 ETP samples, the number of samples harboring each fusion driver was quantified. For each fusion driver, the mean BMP-like AUC, the mean T-specified AUC, the percentage of patients with EOI MRD+, and percentage of patients that died on trial, and the number of unique fusion partners was calculated. Fusion drivers observed in >= 2 samples were plotted for visualization.

### Survival analyses based on gene mutation status

For top T-specified and BMP-like associated genes, 110 ETP patients with corresponding bulk WES/WGS/RNA-seq data were binarized into WT and mutant groups with comparison done by Wilcoxon rank-sum test. Survival analyses were performed using the Cox-Proportional-Hazard test on overall survival. For *NOTCH1* mutated patients, similar analyses were performed by binning patients based on the number of subclonal *NOTCH1* mutations: 0 (WT), 1, or 2+. To compare the fraction of tumor harboring *NOTCH1* mutations, total VAF of corresponding *NOTCH1* mutations was calculated for each patient, with comparison WT, 1 *NOTCH1*-mut, and 2+ *NOTCH1*-mut done by Wilcoxon rank-sum test.

### Identification of a consensus, 17-gene BMP-like gene signature

BMP-like DEGs from ETP-ALL (n=56 BMP-like vs T-specified) and Non-ETP patients (n=445 BMP-like vs Post-Commit) were overlapped and average Log2FC calculated. 17 genes with average Log2FC > 0.9 were retained as a consensus “BMP-17” signature. To contextualize BMP-17 marker genes within normal hematopoiesis, we performed AUC-based signature scoring using AUCell v1.12.0 on healthy donor scRNA-seq reference maps in addition to individually plotting BMP-17 DEGs. To test the clinical significance of the BMP-17 signature, we performed AUC based signature scoring using AUCell v1.12.0 on bulk RNA-sequenced diagnostic T-ALL samples from two independent COG trials (AALL0434, fully sequenced, n = 1051; AALL1231, partially sequenced, n = 75) using BMP-like-17 DEGs. Genes were ranked within each sample using the *AuCell_buildRankings* function and AUC was calculated using AUCell_calcAUC with the top 25% of expressed genes considered in scoring (aucMaxRank = 0.25). We then binarized patients based on AUCell signature score and utilized Cox Proportional Hazard model with EOI MRD and CNS status taken as covariates using the *survfit* function from survival v3.2–13^23^: *survfit(Surv(time.OS, status.OS) ~ BMP17 + high.BMP17 + D29.MRD + cns.status*. In each case, the top 50% of patients were compared with the bottom 50%. Subtype-specific survival analyses were performed by subsetting patients from AALL0434 into: ETP (n=110), Near-ETP (n=168) and Non-ETP (including Non-ETP and Non-Subtyped, n=1057) groups.

### *In silico* drug screening against BMP-like blasts

To identify targetable genes and surface markers that were specific for and necessary to BMP-like blasts, we employed 5 lines of database evidence to nominate top targets amongst 552 BMP-like upregulated DEGs (Log2FC > 0.2; adjusted p-val < 0.01). Drug/target data from two independent drug target databases (TTD^24^, DrugIDB^25^), as well as a third database (OpenTargets^26^) that focuses on next-generation targets were overlapped with BMP-like DEGs. Targetable gene products were given a score of 1 for each database a resulting hit was obtained. To search for drugs that could specifically modify the BMP-like state, we inputted top BMP-like DEGs/TFs (n=56) and top T-specified DEGs/TFs (n=62) into the LINCS1000^27^ database (https:/clue.io/query#l1000) under default parameters. Top DEGs were defined by Log2FC >= 0.25 and adjusted p-value < 0.001 and top TFs by Log2FC >= 0.5 and adjusted p-value <0.01. Perturbation results were filtered using a custom R script which filtered compound-mediated perturbations for compounds with defined targets, statistical significance (log10(FDR) > 1), effect size (normalized connectivity score > 0.8), specificity to BMP-like state (raw connectivity score > 0), and activity in 2 or more leukemia cell lines. Non-compound perturbations, which included ligand-based screens, gene overexpression, shRNA knockdown, and CRISPR knockout were filtered for statistical significance (log10(FDR) > 1) and effect size (normalized connectivity score > 0.8), and further separated into gene overexpression and gene knockdown (including shRNA KD, CRISPR KO, and ligand-based perturbation) classes. Specificity to BMP-like state was defined by positive connectivity score in the case of gene knockdown, and negative connectivity score in the case of gene overexpression. BMP-like DEGs targeted by top compound perturbations and/or genes within gene overexpression/KD were given a score of 1. Finally, to predict BMP-like DEGs that could mediate cytotoxicity when targeted, we downloaded cell line dependency data from the cancer dependency map^28^ (DEPMAP) portal and searched for BMP-like DEGs which showed increased dependency in leukemia cell lines (n=59) compared to non-leukemia/lymphoma cell lines (n=1,052). Genes with negative dependency scores in leukemia cell lines (mean dependency score < −0.1), dependency fold-change > 2, and >25% expression in BMP-like blasts were assigned a score of 1. We further prioritized BMP-like DEGs based on differential expression metrics obtained from single cell data (Average Log2FC, percentage expression, and statistical significance). To prioritize genes with high differential expression, BMP-like DEGs with >1 Log2FC were assigned a score of 1, and genes with Log2FC between 0.5 and 1 were assigned a score of 0.5. Beyond high expression change, we prioritized BMP-like DEGs with high percentage expression in BMP-like blasts: genes with >80% expression (representing near-ubiquitous expression) were assigned a value of 1, whereas genes with 50%−80% expression (representing expression in the majority of blasts) were assigned a value of 0.5. Finally, to prioritize genes that were consistently upregulated in different patients, we ranked genes with high statistical significance (1 = adjusted p-value < 1e-100, 0.5 = adjusted p-value < 1e-50). The sum of DE evidence (ranging from 0–3) and database evidence (ranging from 0–5) was taken to rank BMP-like DEGs for follow-up experimental studies and for visualizations.

### *In vitro* drug screening with leukemia active drug panel

Human leukemia blasts were collected from mouse spleen and enriched using immunomagnetic isolation kit (Stemcell Technologies, #19849) and screened with a panel of 40 leukemia active drugs (Supplementary Table 18) using a previously described imaging-based assay with a stromal cell co-culture system^29^.

### Nomination of BMP-like specific drugs from *in vitro* drug screening

PDX expanded blasts from 5 patients were screened in a stromal co-culture system and dose response curves were generated for each compound with the primary readout being cell viability (% of control). We defined ETP active drugs as compounds with IC50 < 1000nM and categorized each compound as not active (active in 0/4 ETP patients), partially active (1–3 ETP patients), or active (4/4 ETP patients). We then compared IC50 values for ETP active compounds and utilized three comparisons to nominate drugs that were differentially active in High BMP patients: ETP BMP-high/MRD+ (n=3) vs BMP-low/MRD− (n=2, one ETP, one Non-ETP); BMP-high/HighMRD (n=2) vs ETP BMP-low (n=1); BMP-high/MRD+ (n=3) vs ETP BMP-low (n=1). Drugs with differential activity in BMP-high patients in all three comparisons were nominated as BMP-specific drugs.

## Data Availability

Raw sequencing data for this study are in preparation for submission to dbGaP (accession number pending).
